# Limb-Sparing Resection with Prophylactic Tibial Fixation and Medial Gastrocnemius Flap Reconstruction for Recurrent Leiomyosarcoma in a Previously Irradiated Leg: A Case Report

**DOI:** 10.3390/reports9030298

**Published:** 2026-09-04

**Authors:** Georgi P. Luchev, Lyubomir Gaydarski, Svetoslav A. Slavchev, Ahmed Al-Sadek, Vera Megdanova, Iva N. Dimitrova, Georgi P. Georgiev

**Affiliations:** 1 Department of Orthopedics and Traumatology, University Hospital Queen Giovanna-ISUL, Medical University of Sofia, 1527 Sofia, Bulgaria; georgi.luchev06@gmail.com (G.P.L.); s.slavchev@medfac.mu-sofia.bg (S.A.S.); aalsadek@medfac.mu-sofia.bg (A.A.-S.); 2 Department of Anatomy, Histology and Embryology, Medical University of Sofia, 1431 Sofia, Bulgaria; lgaydarski@medfac.mu-sofia.bg; 3 Department of Medical Oncology, University Hospital Queen Giovanna-ISUL, Medical University of Sofia, 1527 Sofia, Bulgaria; megdanova.vera@abv.bg; 4 Department of Cardiology, University Hospital “St. Ekaterina”, Medical University of Sofia, 1431 Sofia, Bulgaria; dimytrova@yahoo.com

**Keywords:** leiomyosarcoma, local recurrence, limb-sparing surgery, prophylactic fixation, locking compression plate, medial gastrocnemius flap, irradiated field

## Abstract

**Background and Clinical Significance**: Local recurrence of extremity leiomyosarcoma after multiple operations and radiotherapy presents a major therapeutic challenge. Adequate oncologic clearance must be balanced against preservation of skeletal stability and reliable wound coverage within a scarred and poorly vascularized tissue bed; **Case presentation**: A 78-year-old woman presented with a painful recurrent leiomyosarcoma of the anterior proximal third of the right leg after three previous operations and adjuvant radiotherapy. Preoperative magnetic resonance imaging demonstrated a recurrent soft-tissue lesion extending to the anterior surface of the proximal tibia. Staging computed tomography of the chest, abdomen, and pelvis showed no distant metastatic disease before definitive surgery. A one-stage limb-sparing procedure was performed, including resection of the recurrent tumor bed and an approximately 7-cm anterior cortical lamella of the proximal tibia containing an area considered suspicious for neoplastic involvement. A locking tibial plate was applied prophylactically to reduce the risk of pathological fracture. The approximately 12 × 10-cm soft-tissue defect was covered with a muscle flap from the medial head of the gastrocnemius and a free skin graft harvested from the paraumbilical region. Gross pathological examination identified an 8-cm subcutaneous tumor formation. Histopathological examination demonstrated well-differentiated leiomyosarcoma; the spindle cells expressed vimentin, actin, and desmin. All examined resection lines were free of tumor infiltration. No pathogenic microorganism was isolated, and no early postoperative complication was documented. The patient mobilized with walking aids and protected weight bearing for 30 days. At approximately 16 months, the flap and skin graft provided stable coverage, no postoperative tibial fracture or clinically apparent implant-related complication had occurred, and the patient was independently ambulatory. Postoperative MRI demonstrated expected postoperative changes and a small indeterminate subcutaneous focus without diffusion restriction, requiring continued surveillance; **Conclusions**: This case illustrates the feasibility of combining oncologic resection, prophylactic tibial stabilization, and vascularized soft-tissue reconstruction in a previously irradiated extremity. The documented early functional and reconstructive outcome supports this individualized limb-sparing approach, although longer oncologic surveillance is required.

## 1. Introduction and Clinical Significance

Leiomyosarcoma is a malignant mesenchymal neoplasm exhibiting smooth muscle differentiation that can arise in the uterus, retroperitoneum, blood vessels, trunk, or extremities. Somatic extremity leiomyosarcomas are uncommon and frequently present as slowly enlarging, nonspecific soft-tissue masses, requiring accurate histopathological diagnosis, comprehensive staging, and multidisciplinary treatment planning [1,2,3]. For localized disease, the principal treatment is complete surgical excision with microscopically negative margins, as inadequate local clearance significantly elevates the risk of local failure. Overall prognosis and recurrence risk are dictated by several clinicopathological factors, with high histological grade and increasing tumor size being particularly associated with distant recurrence and disease-specific mortality [4,5,6,7,8].

The clinical management of these tumors is frequently complicated by initial unplanned excisions. Such procedures disrupt anatomical planes, contaminate the operative bed, and often leave microscopic or macroscopic residual disease. While the impact of an unplanned excision on overall survival remains variable, the presence of residual tumor at re-excision is consistently linked to impaired local control, necessitating much larger definitive resections and more extensive reconstructive procedures [9,10,11,12]. These surgical challenges are exceptionally magnified when recurrent disease develops in a tissue bed previously subjected to both repeated operations and adjuvant radiotherapy. Prior irradiation severely impairs local vascularity and tissue elasticity, amplifying the risks of wound dehiscence, infection, delayed healing, and radiation-associated bone injury. In these salvage scenarios, achieving adequate oncologic clearance may require the removal of a substantial cortical bone segment, which in turn necessitates prophylactic skeletal stabilization to prevent pathological fracture. Furthermore, in regions such as the proximal leg, robust vascularized muscle coverage is essential to protect the exposed bone and fixation hardware, ensuring a reliable recipient bed for subsequent skin grafting [13,14,15].

To illustrate a successful multidisciplinary approach to these compounded clinical challenges, we report the treatment of a recurrent proximal leg leiomyosarcoma in a 78-year-old woman who had previously undergone three operations and radiotherapy. This case highlights the technical feasibility and functional outcome of a comprehensive, one-stage salvage procedure combining tumor-bed resection, anterior tibial cortical lamellar resection, prophylactic locking-plate fixation, and medial gastrocnemius muscle flap reconstruction.

## 2. Case Presentation

### 2.1. Patient History and Clinical Presentation

A 78-year-old woman presented with a painful, progressively enlarging recurrent lesion over the anterior aspect of the proximal third of her right leg. Her medical history was significant for type 2 diabetes mellitus managed with metformin and arterial hypertension on combination therapy. She had no family history of cancer or known medication allergies.

The patient’s orthopedic oncologic history began approximately six years prior to her current presentation when she first noticed swelling in the right proximal leg. She underwent an initial unplanned excision at a secondary hospital, followed closely by a re-excision one month later after receiving a histopathological diagnosis of leiomyosarcoma. A subsequent local recurrence necessitated a third operation at a tertiary center eight months after the initial procedure. Following this third resection, she received adjuvant external-beam radiotherapy to the tumor bed, delivering a total dose of 56 Gy in 28 fractions (2 Gy/fraction) over the subsequent two months.

The patient remained under surveillance for approximately five years until she developed new progressive swelling and pain within the fibroatrophic and irradiated operative field. Clinical examination revealed a recurrent focal mass associated with compromise of the overlying skin and minimal serous discharge. There were no clinical signs of systemic infection, and distal neurovascular status remained intact with preserved perfusion and no motor or sensory impairments.

### 2.2. Diagnostic Imaging and Preoperative Staging

**Figure 1:**
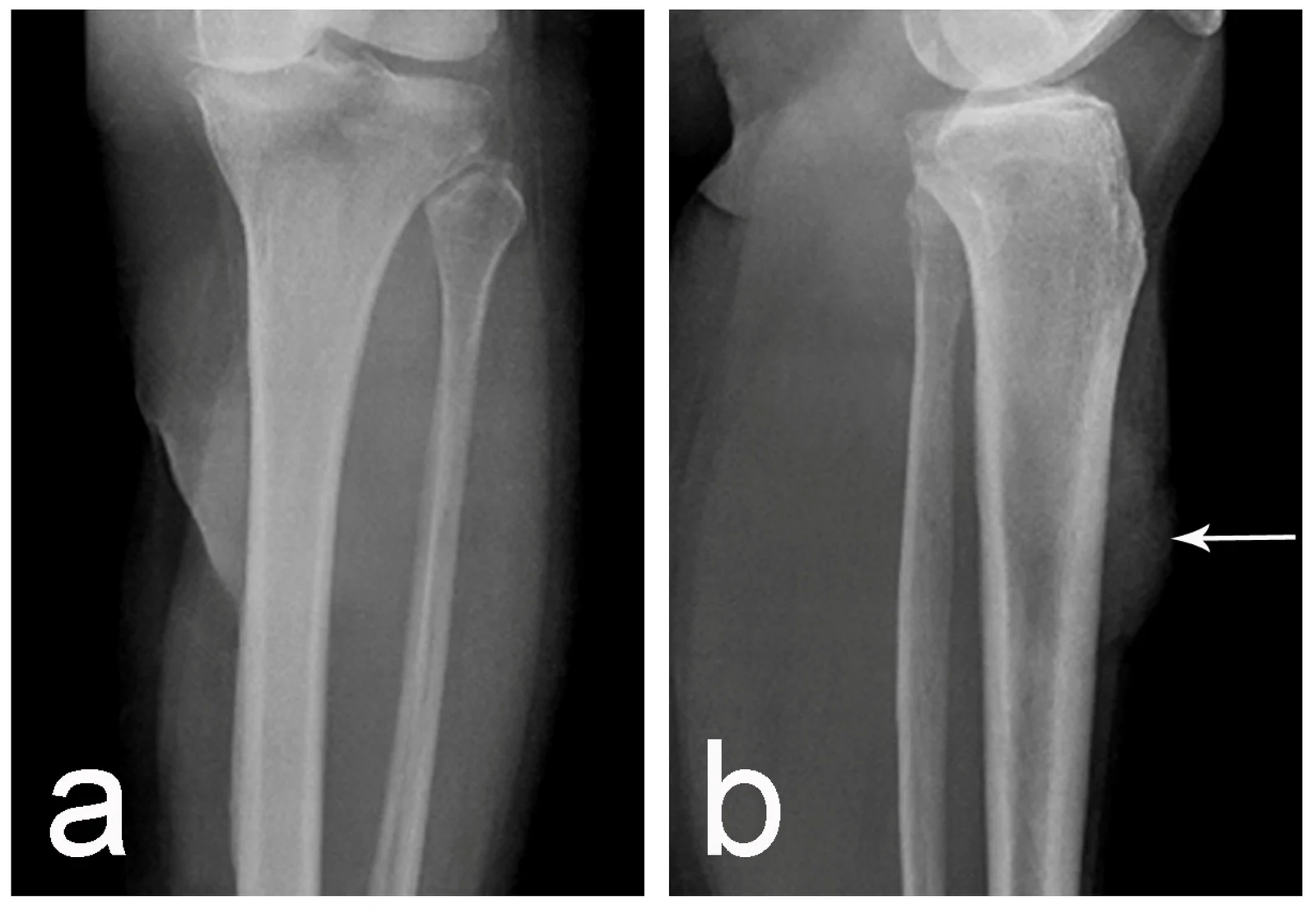
Preoperative anteroposterior (**a**) and lateral (**b**) radiographs of the proximal tibia demonstrating the baseline osseous condition before definitive resection as well as the soft-tissue component (arrow).

Baseline anteroposterior and lateral radiographs documented the osseous condition of the proximal tibia [Figure 1].

**Figure 2:**
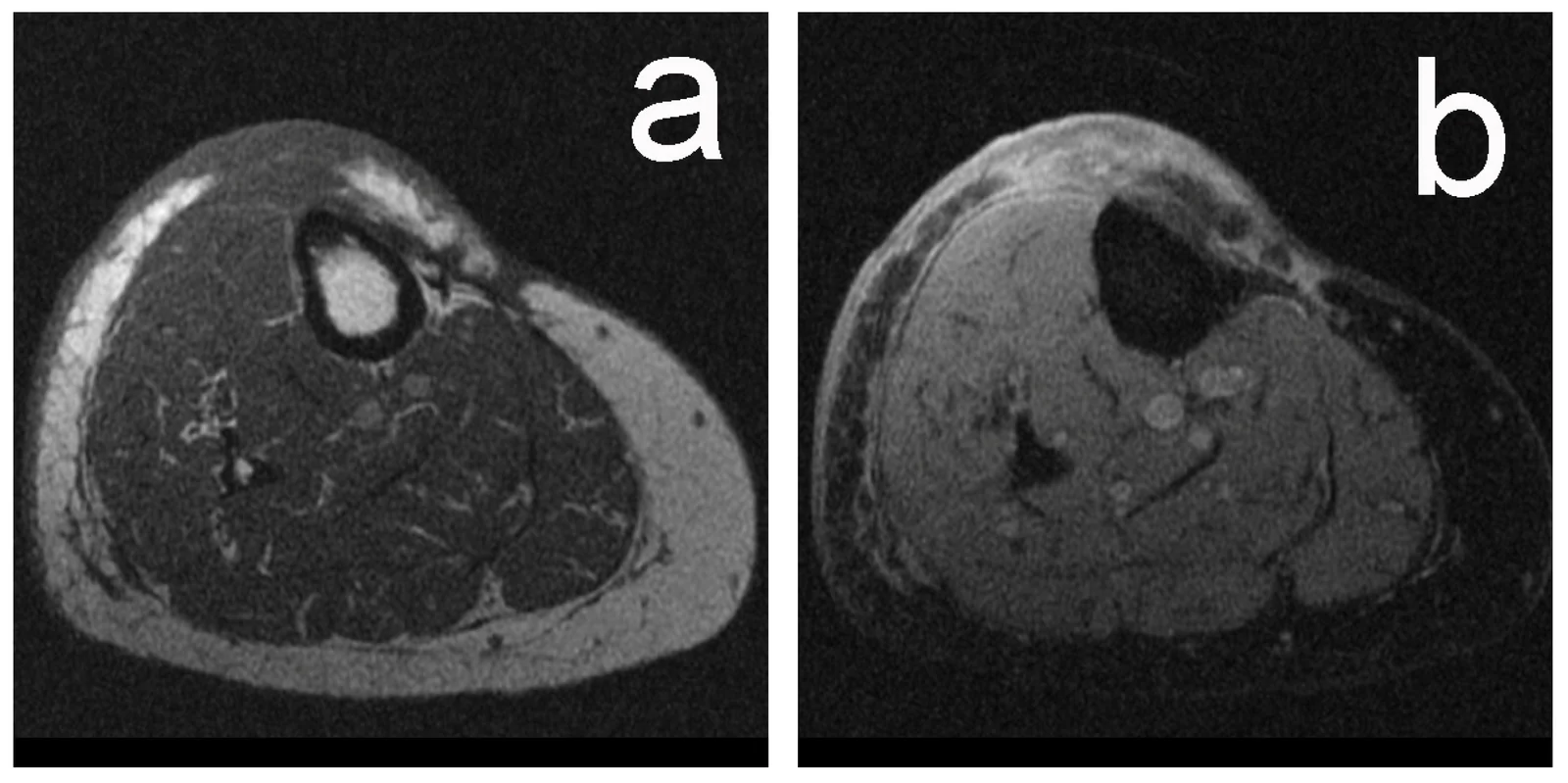
Preoperative contrast-enhanced MRI (**a**,**b**) of the right leg demonstrating the recurrent soft-tissue lesion extending through the anterior soft tissues to the anterior surface of the proximal tibia.

A preoperative contrast-enhanced MRI of the right leg demonstrated a recurrent soft-tissue lesion extending through the anterior soft tissues and directly abutting the anterior surface of the proximal tibia [Figure 2]. A core-needle biopsy performed preoperatively confirmed recurrent well-differentiated leiomyosarcoma. Subsequent preoperative staging CT of the chest, abdomen, and pelvis showed no evidence of distant metastatic dissemination.

### 2.3. Treatment Strategy and Surgical Intervention

Because the recurrence was localized and surgically resectable, a one-stage limb-sparing approach was selected. Systemic chemotherapy was omitted from the neoadjuvant and adjuvant treatment plan due to conflicting literature regarding its benefit for low-grade leiomyosarcomas [16,17]. The multidisciplinary surgical objectives were en bloc resection of the recurrent tumor bed, prophylactic skeletal stabilization, and robust soft-tissue coverage of the compromised, irradiated field.

**Figure 3:**
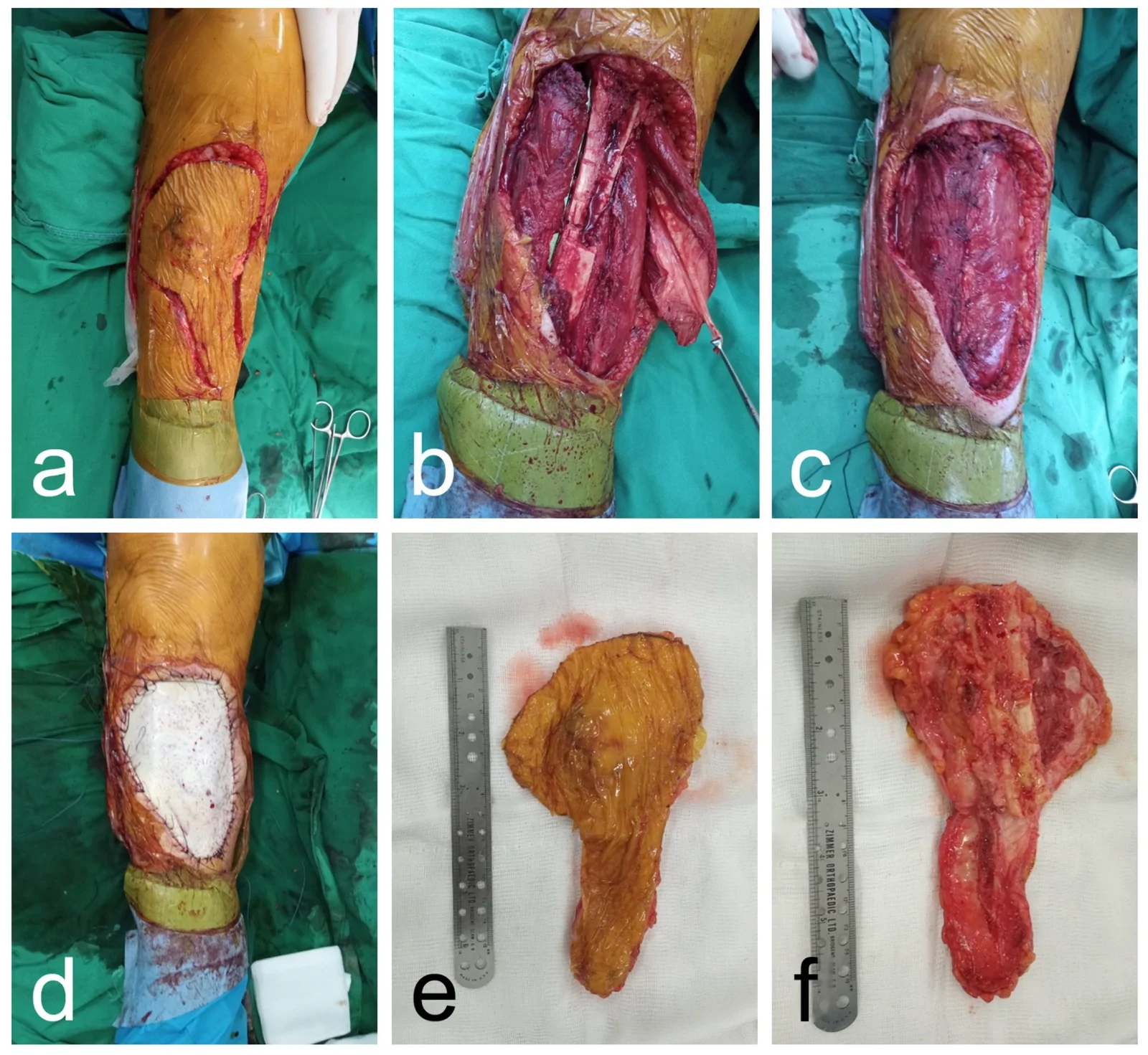
Sequential intraoperative views demonstrating the surgical progression. (**a**) Circumferential skin incision encompassing all previous surgical scars. (**b**) Tumor bed resection and prophylactic tibial plating. (**c**) Robust soft-tissue reconstruction achieved utilizing a medial gastrocnemius muscle flap and (**d**) free skin graft. (**e**) Superficial aspect of resected specimen. (**f**) Deep aspect of resected specimen showing en bloc resection of bone and soft tissues.

The operation proceeded shortly thereafter with the patient positioned supine. After obtaining negative preoperative swab cultures from the ulceration, a skin flap was designed to incorporate the prior cicatricial tissue. Sharp dissection was carried down through the subfascial structures, and an approximately 7-cm anterior cortical lamella of the proximal tibia—encompassing the area suspicious for neoplastic involvement—was resected and submitted for pathological evaluation. To mitigate the risk of a pathological fracture following this cortical resection, prophylactic internal stabilization was achieved using a locking tibial plate spanning the defect. The resulting 12 × 10 cm soft-tissue void was reconstructed utilizing a pedicled muscle flap from the medial head of the gastrocnemius. The remaining cutaneous defect was covered with a free skin graft harvested from the paraumbilical region [Figure 3]. The donor site was closed in layers, and the limb was dressed with a sterile compressive bandage.

### 2.4. Histopathological Findings

**Figure 4:**
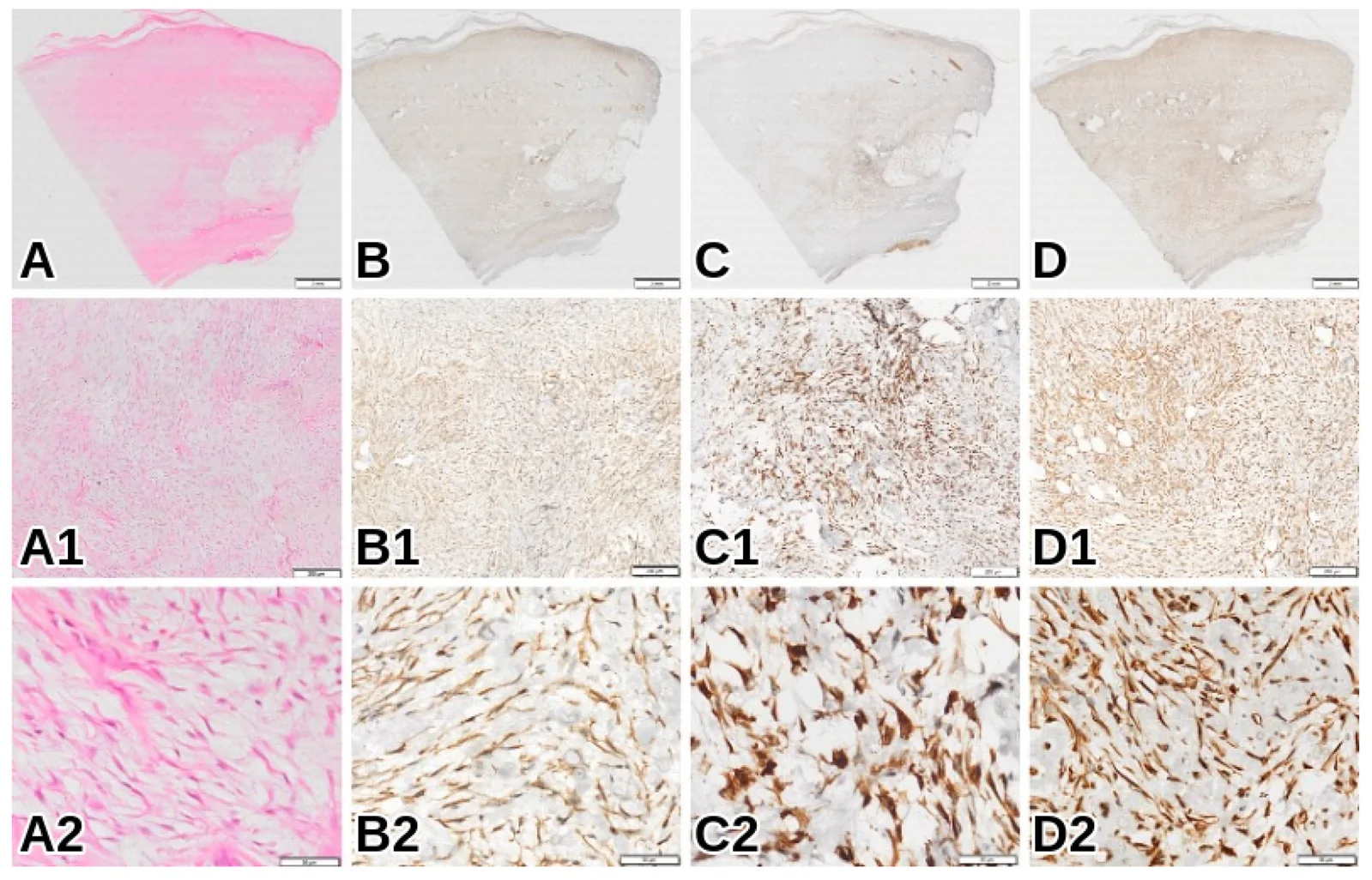
Histopathological and immunohistochemical profile of the recurrent proximal leg leiomyosarcoma. (**A**–**D**) Low-power overview (scale bars = 2 mm) demonstrating the densely cellular subcutaneous tumor mass beneath the overlying skin. (**A**) Hematoxylin and eosin (H&E) staining reveals a solid mesenchymal neoplasm. The tumor demonstrates diffuse, strong macroscopic positivity for (**B**) smooth muscle actin (SMA), (**C**) desmin, and (**D**) vimentin. (**A1**–**D1**) Intermediate magnification (scale bars = 200 µm) highlighting the characteristic architecture of intersecting spindle cell fascicles. (**A1**) H&E stain. (**B1**–**D1**) Robust and uniform cytoplasmic expression of SMA, desmin, and vimentin is evident throughout the fascicular network, confirming smooth muscle lineage. (**A2**–**D2**) High-power magnification (scale bars = 50 µm) detailing the cytomorphology. (**A2**) Spindle cells exhibit abundant, deeply eosinophilic cytoplasm and characteristic blunt-ended, cigar-shaped nuclei with moderate pleomorphism. (**B2**–**D2**) High-power confirmation of intense cytoplasmic reactivity for SMA, desmin, and vimentin.

Gross pathological evaluation of the 15 × 17 cm composite resection specimen (skin, musculature, and bone) confirmed an 8-cm subcutaneous tumor. Microscopically, hematoxylin and eosin (H&E) stained sections revealed a densely cellular mesenchymal neoplasm situated beneath an area of dense collagenous fibrosis, correlating with the clinically noted keloid scar. The tumor was composed of intersecting fascicles of spindle cells exhibiting abundant, deeply eosinophilic cytoplasm and characteristic blunt-ended, cigar-shaped nuclei with moderate nuclear pleomorphism [Figure 4(A2)].

Immunohistochemical (IHC) staining was performed on formalin-fixed, paraffin-embedded (FFPE) tissue sections using standard heat-induced epitope retrieval methods. The neoplastic spindle cells demonstrated diffuse and strong positive cytoplasmic expression for smooth muscle actin (SMA), desmin, and vimentin [Figure 4(B1–D1)]. This robust immunophenotypic profile definitively confirmed the smooth muscle lineage of the tumor, yielding a final diagnosis of well-differentiated leiomyosarcoma. While all examined resection lines were free of tumor infiltration, the pathology report did not specify the FNCLCC grade, mitotic count, percentage of tumor necrosis, exact margin distances, or the definitive histological relationship between the tumor and the resected tibial cortex.

### 2.5. Postoperative Course and Follow-Up

The patient’s early postoperative recovery was entirely uncomplicated, with no evidence of surgical-site infection, wound dehiscence, flap compromise, or implant-related complications. The gastrocnemius muscle flap remained viable, and the free skin graft provided stable coverage. She was mobilized using walking aids with protected weight-bearing for 30 days before transitioning to progressive loading.

**Figure 5:**
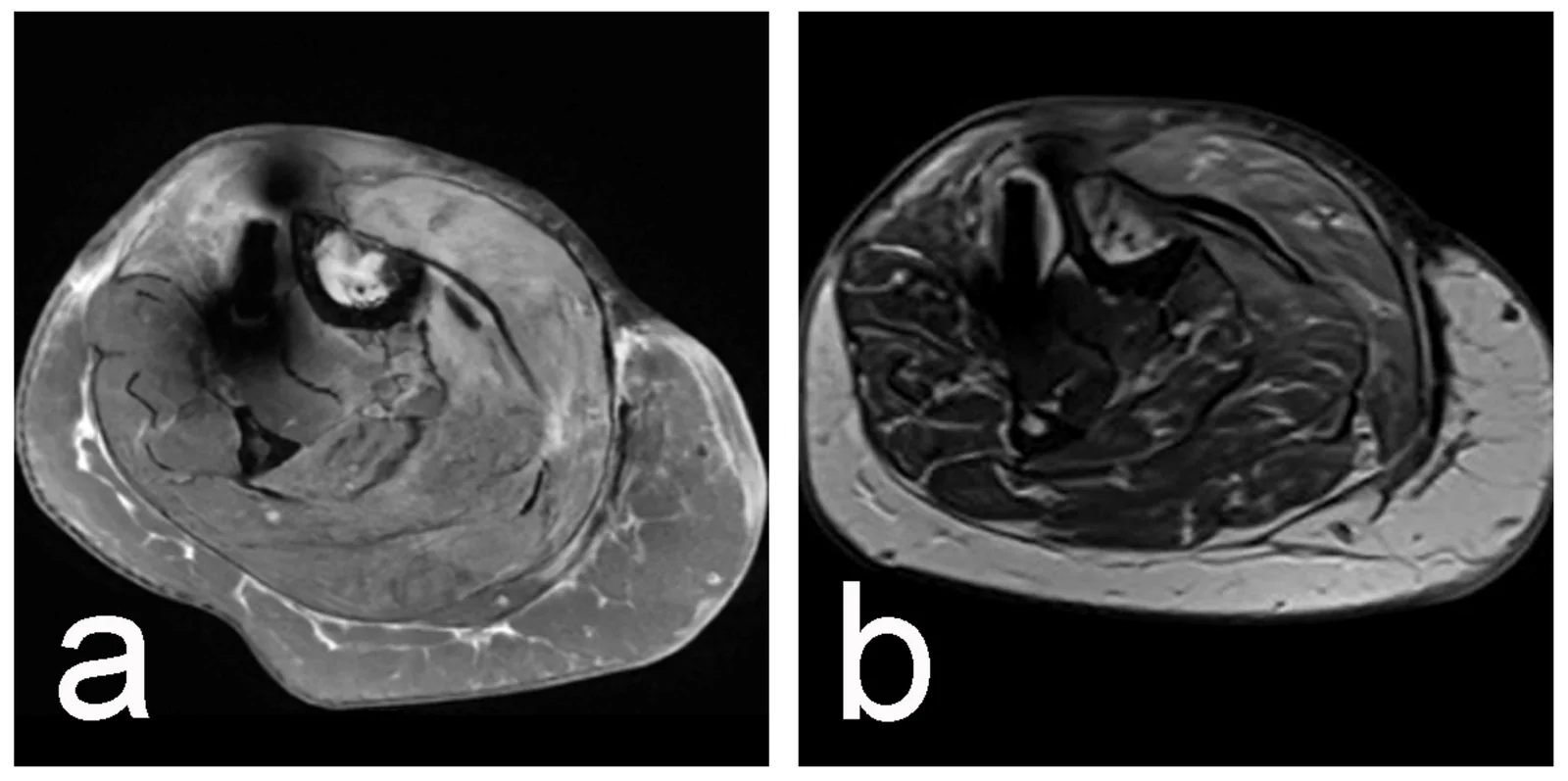
Postoperative contrast-enhanced MRI (**a**,**b**) showing the anteromedial tibial cortical defect, plate-and-screw stabilization, and reconstructed soft tissues.

**Figure 6:**
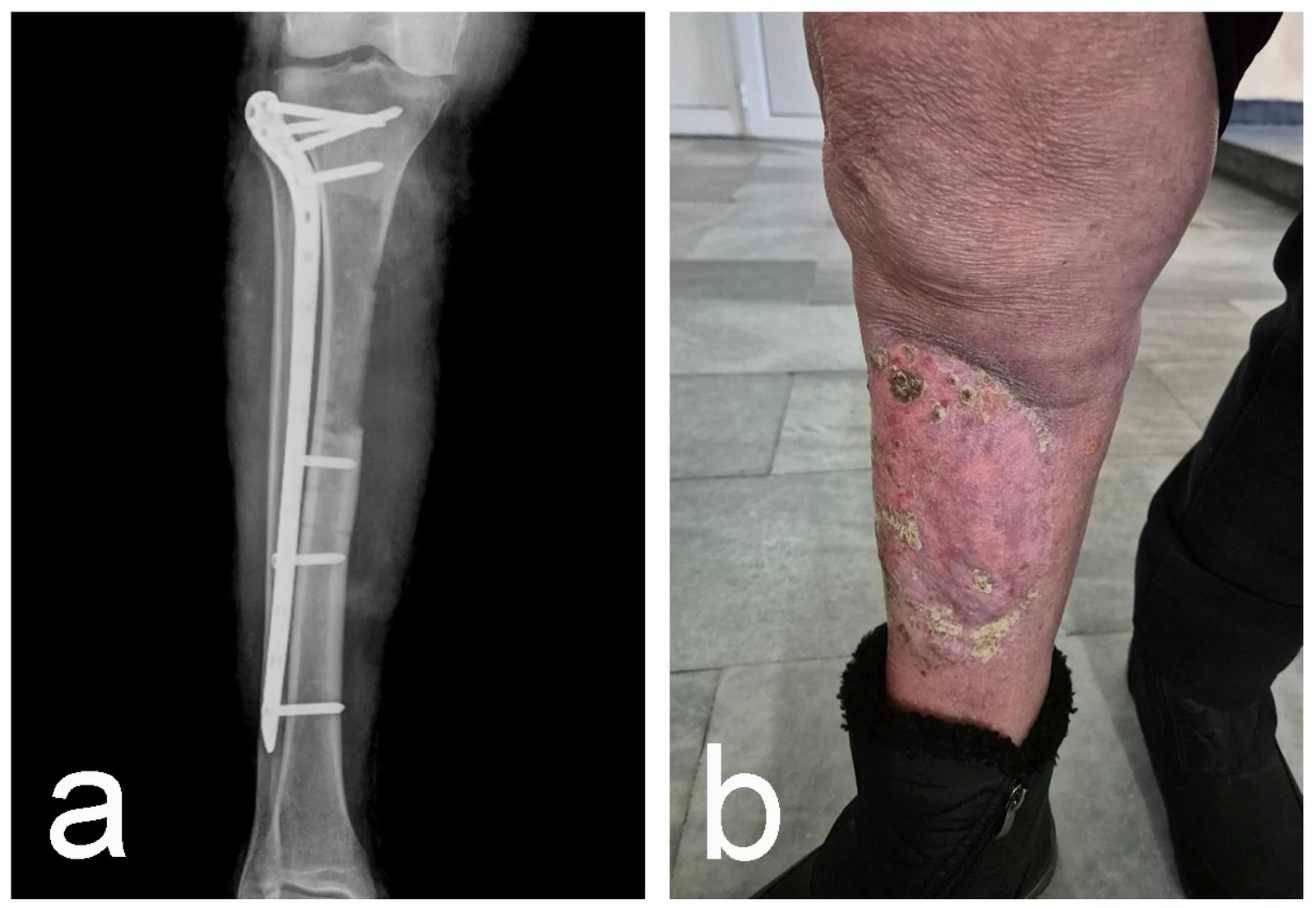
Postoperative imaging and clinical outcome at approximately 16 months. (**a**) Anteroposterior radiograph demonstrating the prophylactic locking tibial plate. (**b**) Clinical photograph showing stable coverage by the gastrocnemius muscle flap and free skin graft without wound breakdown or hardware exposure.

A follow-up contrast-enhanced MRI at nine months postoperatively demonstrated expected postoperative changes, including the anteromedial tibial cortical defect supported by plate-and-screw stabilization [Figure 5 and Figure 6a]. The osseous defect measured approximately 100 mm on imaging, representing a minor discrepancy from the 7-cm measurement documented in the operative report. The medullary cavity showed a heterogeneous signal without restricted diffusion. However, a small (10 × 9 × 4 mm) spindle-shaped subcutaneous lesion with peripheral contrast enhancement was noted on the medial leg, 88 mm distal to the joint line. Lacking diffusion restriction, this focus was deemed indeterminate and marked for continued surveillance.

At her 16-month follow-up, the patient was independently ambulatory without assistive devices and had returned to her normal activities of daily living. Clinical examination confirmed stable, durable coverage by the gastrocnemius flap and skin graft, with no hardware exposure or wound breakdown [Figure 6b]. She reported high satisfaction with the preservation of her limb and maintenance of her independence. No clinically evident local or systemic disease progression has been documented, and the indeterminate MRI focus continues to be actively monitored.

## 3. Discussion

This case illustrates the complexity of treating recurrent extremity leiomyosarcoma after multiple previous operations and radiotherapy. Limb preservation required management of three interrelated problems: resection of the recurrent tumor bed, prophylactic stabilization after partial tibial cortical resection, and soft-tissue coverage within irradiated tissues.

Leiomyosarcoma is an aggressive soft-tissue sarcoma with clinically heterogeneous behavior. Tumor size, depth, and histological grade are consistently associated with outcome. Gladdy et al. found tumor size and grade to be independent predictors of disease-specific survival and distant recurrence, whereas size and surgical margin status influenced local recurrence. Late recurrence may occur even after an initially prolonged disease-free interval, supporting the need for long-term surveillance [5]. In a consecutive series of somatic leiomyosarcomas, depth and high histological grade were also associated with an adverse prognosis [4]. Harati et al. similarly identified histological grade as a major determinant of disease-specific survival in surgically treated somatic soft-tissue leiomyosarcoma [18]. More recently, a machine-learning model incorporating tumor size, grade, and location was developed to predict 5-year survival in soft-tissue leiomyosarcoma [7], and a single-center genomic analysis identified ATRX mutation status and pleural metastases as additional independent predictors of overall survival [8]. The patient’s treatment history also demonstrates the challenges that may follow an initial unplanned sarcoma excision. Such procedures are performed without appropriate preoperative imaging, biopsy planning, or margin-oriented resection. They may leave residual tumor and disturb additional anatomical planes, thereby increasing the complexity of definitive tumor-bed resection. Potter et al. observed high rates of residual disease and increased local recurrence after unplanned excision of high-grade soft-tissue sarcomas, despite subsequent tumor-bed resection [9]. Systematic reviews have confirmed that residual tumor is frequently identified during re-excision and is associated with poorer local recurrence-free survival [10,11]. The relationship between an initial unplanned excision and subsequent outcome is not deterministic. Tumor biology, grade, size, depth, residual disease, and the quality of subsequent treatment all influence the final outcome. In the present case, the available documentation confirms multiple previous procedures before the definitive operation but does not establish the individual contribution of each procedure to the later recurrence. An important feature of the definitive operation was resection of an approximately 7-cm anterior cortical lamella of the proximal tibia that included an area considered suspicious for neoplastic involvement. The pathology report confirmed that the resection specimen contained underlying bone but did not separately document histological invasion of the periosteum or cortex. The cortical resection prompted prophylactic fixation with a locking tibial plate to reduce the risk of pathological fracture. Evidence defining a precise biomechanical threshold for prophylactic fixation after limited tibial cortical resection is sparse. However, given that the approximately 7-cm anterior cortical defect represented a substantial structural compromise in a previously irradiated and mechanically vulnerable bone segment, immediate stabilization was deemed clinically necessary. No postoperative fracture or clinically apparent implant-related complication was documented during approximately 16 months of follow-up. A single case cannot establish general criteria for prophylactic fixation, and longer follow-up remains necessary. Soft-tissue reconstruction was an integral component of the operation. A muscle flap from the medial head of the gastrocnemius was used to cover the defect, followed by a free skin graft from the paraumbilical region. The medial gastrocnemius flap is a well-established reconstructive option for defects involving the proximal tibia and knee and has been used to cover implants and exposed structures in oncologic reconstruction [6,13,14,15,19,20,21]. A comparative summary of the reconstructive and prophylactic strategies utilized in the present case alongside similar reports from the literature is outlined in Table 1.

No early wound, flap, graft, or implant-related complication was documented. At follow-up, the flap and graft continued to provide stable soft-tissue coverage. Post-treatment imaging of a sarcoma bed may be difficult to interpret because postoperative edema, fibrosis, granulation tissue, and enhancement can overlap with the appearance of recurrent disease. The small peripherally enhancing subcutaneous focus identified on MRI lacked diffusion restriction and was classified as indeterminate. Continued surveillance was recommended. The role of systemic treatment in completely resectable localized extremity leiomyosarcoma is individualized. In patients with moderate- and high-grade leiomyosarcoma, results regarding a perioperative benefit in overall survival (OS) and relapse-free survival (RFS) are limited, depending on the trials in the last decade [17]. Taking this information into consideration for patients like ours with G1 LMS, we do not expect better results from perioperative chemotherapy for local and systemic control in the case of a localized operable recurrence. In the present patient, preoperative CT staging showed no distant metastatic disease, and a limb-sparing surgical procedure was performed.

### Limitations

The present case report has several limitations. First, it describes a single patient and cannot establish the superiority of the selected reconstruction or fixation strategy over alternative methods. Second, the available pathology report did not include an FNCLCC grade, mitotic count, percentage of tumor necrosis, exact margin distances, or a separate assessment of periosteal or cortical invasion. Future retrospective re-evaluation of the archived histological sections utilizing digital image analysis software, such as Fiji/ImageJ, to accurately map and quantify the margin distances and periosteal interface could resolve this data gap. Third, complete pathological and margin data from the operations performed in 2019 were not available for direct comparison with the recurrent lesion. Fourth, the exact dimensions and circumferential extent of the cortical resection were not explicitly documented. The discrepancy between the operative report (an approximately 7-cm resection) and the postoperative MRI measurement (a 100-mm defect) is likely attributable to imaging artifacts generated by the metallic locking plate or differing measurement landmarks utilized during the surgical procedure versus the radiological assessment. Fifth, the indication for prophylactic fixation was based on surgical judgment rather than a validated biomechanical threshold. Sixth, functional outcome was assessed using clinical observations and independent ambulation rather than a validated musculoskeletal oncology score. Seventh, a small indeterminate subcutaneous focus identified on postoperative MRI requires ongoing surveillance. Finally, no follow-up systemic staging result was available for assessment of distant disease status at the latest clinical follow-up. The documented outcome should therefore be interpreted as evidence of technical feasibility and early functional success rather than definitive long-term oncologic control.

## 4. Conclusions

Recurrent leiomyosarcoma in a multiply operated and irradiated extremity requires coordinated oncologic, orthopedic, and reconstructive planning. In this patient, resection of the recurrent tumor bed and an approximately 7-cm anterior tibial cortical lamella, prophylactic locking-plate fixation, and medial gastrocnemius muscle flap coverage resulted in resection lines free of tumor infiltration, stable wound coverage, and preservation of independent ambulation during approximately 16 months of follow-up. The case supports the feasibility of a one-stage limb-sparing approach when tibial cortical resection is combined with prophylactic stabilization and soft-tissue reconstruction. It does not establish universal criteria for prophylactic fixation. Continued local and systemic surveillance remains necessary.

## 5. Patient

At the latest clinical follow-up, the patient reported satisfaction with preservation of the affected limb and remained independently ambulatory without assistive devices.

## Figures and Tables

**Table 1:** Summary of Reconstructive and Prophylactic Strategies for Extremity Sarcomas and Proximal Tibial Defects.

Author & Year	Number of Patients	Pathology/Tumor Type	Prior Radiation	Osseous Defect/Resection	Prophylactic Fixation	Soft-Tissue Coverage	Follow-Up & Outcome
Current Case	1	Recurrent leiomyosarcoma (G1)	Yes (56 Gy)	7-cm anterior tibial cortical lamella	Yes (Locking tibial plate)	Medial gastrocnemius muscle flap + free skin graft	16 months; stable coverage, independently ambulatory
Buchner et al., 2003 [13]	25	Malignant bone tumors (predominantly osteosarcoma)	Not specified (20 patients received neoadjuvant chemotherapy, 1 received postoperative radiotherapy)	Proximal tibia resection (distal femur also resected in 2 patients)	Modular, constrained total-knee endoprosthesis (18 patients) or allograft/autograft reconstruction (7 patients)	Medial gastrocnemius muscle flap (24 patients) or lateral head (1 patient); secondary split-thickness skin graft in 13 patients	Mean 4 years 6 months; 96% flap survival without complications; mean MSTS score 75.1%; 96% limb salvage rate
El-Sherbiny, 2008 [14]	30	Localized bone or soft tissue sarcoma around the knee and popliteal fossa	Not specified (Adjuvant chemotherapy or radiotherapy given postoperatively per protocol)	Wide resection of proximal tibia or distal femur	Endoprosthetic reconstruction	Medial gastrocnemius muscle flap (21 cases) or myocutaneous gastrocnemius flaps (8 cases); required donor site grafting	Mean 52 months; 100% flap survival; 96.6% limb salvage success rate; high MSTS functional scores
Walton et al., 2017 [15]	N/A (Review Article/Surgical Technique)	Various (tumors, trauma, infections, extensor mechanism discontinuity)	N/A	Proximal third of the tibia or knee joint	Megaprostheses, total knee arthroplasties, or exposed hardware	Pedicled rotational medial and lateral gastrocnemius flaps	N/A (Review article; notes generally good functional results and low failure rates across literature)

## Data Availability

The relevant clinical data are presented within the article. Additional anonymized information may be made available by the corresponding author upon reasonable request and subject to applicable patient-privacy restrictions.

## Abbreviations

The following abbreviations are used in this manuscript:

CT	Computed Tomography
FFPE	Formalin-Fixed, Paraffin-Embedded
FNCLCC	Fédération Nationale des Centres de Lutte Contre le Cancer (French Federation of Cancer Centers)
G1	Grade 1
Gy	Gray (Unit of radiation dose)
H&E	Hematoxylin and Eosin
IHC	Immunohistochemical/Immunohistochemistry
LMS	Leiomyosarcoma
MRI	Magnetic Resonance Imaging
MSTS	Musculoskeletal Tumor Society
OS	Overall Survival
RFS	Relapse-Free Survival
SMA	Smooth Muscle Actin
